# Socio-behavioral factors, oral hygiene level and periodontitis prevalence in a 35-44-year-old Greek adult population: A cross-sectional survey

**DOI:** 10.4317/jced.58507

**Published:** 2021-10-01

**Authors:** Iliana Diamanti, Argy Polychronopoulou, William Papaioannou, Charis Theodoridis, Vasileios Margaritis, Eleni Mamai-Homata, Sotirios Kalfas

**Affiliations:** 1 Department of Preventive and Community Dentistry, School of Dentistry, National and Kapodistrian University of Athens, Athens, Greece; 2 Department of Preventive Dentistry, Periodontology and Implant Biology, School of Dentistry, Aristotle University of Thessaloniki, Thessaloniki, Greece; 3 School of Health Sciences, Walden University, Minneapolis, Minnesota, USA

## Abstract

**Background:**

Sub-optimal oral hygiene is considered as a primary risk factor for periodontitis occurrence. Various socio-behavioral determinants may contribute either independently or by influencing adversely the oral hygiene (OH) level. The aim of the present study was to examine the periodontal status of 35-44-year-old Greek adults and determine the contribution of risk indicators, including the socio-behavioral aspects and the population’s oral hygiene level, on disease prevalence.

**Material and Methods:**

In 1218 participants, Community Periodontal Index (CPI), Loss of Attachment (LoA) and simplified Oral Hygiene Index (OHI-S) were calculated. Multivariable regression models examined the effect of socio-behavioral factors with/without the inclusion of OHI-S level on pocket depth (PD)≥4mm and LoA≥4mm presence.

**Results:**

11.8% of the participants had healthy periodontium, whereas 37.3% and 5.6% presented with shallow and deep pocketing, respectively. 60.4%, 28.8%, and 10.8.% of the adults demonstrated LoA≤3mm, 4-5mm, and ≥6mm, accordingly. Fair and poor oral hygiene significantly increased the likelihood for PD≥4mm (OR=4.8-20.3) and LoA≥4mm (OR=3.3-6.0) presence. ‘Emergency-oriented dental visiting pattern’ significantly elevated the chance for PD≥4mm presence (OR=1.7). ‘Lower education level’ ‘urban location’, and ‘using an interdental brush’ were significantly independently linked to LoA≥4mm occurrence (OR=1.7-2.1, 1.5, and 2.0, respectively). Lower educated individuals demonstrated inferior oral hygiene status, which in turn elevated significantly the chances of PD≥4mm presence. Smoking more than 10 cigarettes/day, emergency-oriented dental attendance pattern and not flossing were linked to worse oral hygiene levels, which consequently increased significantly the likelihood of LoA≥4mm occurrence.

**Conclusions:**

Fair and poor oral hygiene contribute strongly to periodontitis occurrence. Various socio-behavioral factors may influence adversely oral hygiene maintenance, leading to periodontitis manifestations.

** Key words:**Community periodontal index, periodontal attachment loss, oral hygiene, adults 35-44, cross-sectional survey, socio-behavioral indicators.

## Introduction

Periodontal disease is recognized as a major oral pathological condition, reported as being a common entity among adult populations worldwide (1), although its quantification in a reproducible and comparable manner has been an ongoing challenge (2). It is reported to impact adversely the oral health-related quality of life of patients (3), and may lead to tooth loss with associated multidimensional impairment (4). Furthermore, periodontitis is well documented as a major contributor to systemic inflammation burden of the individual, which is strongly implicated in coronary artery disease, stroke and Type II diabetes (5). Also, the evidence regarding possible direct distal effects of periodontal bacteremia on adverse pregnancy outcomes, implies a significant association (6). Therefore, the estimation and understanding of periodontal disease occurrence at the population-level is regarded as highly important for all healthcare providers (7).

Similar to general health, profound disparities have been reported among populations concerning periodontal health (7). This variance may result from differences in examination protocols and periodontitis case definitions among studies, however, differences in demographic characteristics and levels of exposure to various risk factors among populations are thought to play a major role (1). Therefore, when evaluating periodontal disease manifestation in population field studies, risk analysis has to be performed (8). Related factors include demographic indicators such as gender (9), socio-economic position parameters, particularly education and income (10,11), oral hygiene behaviors such as toothbrushing frequency (12) and the use of interdental cleaning devices (13), dental attendance patterns (14) and lifestyle dimensions such as smoking habits (15). Furthermore, clinical indicators, such as the oral hygiene level, have been strongly linked to periodontitis occurrence (16). Specifically, current evidence suggests a dose-response relationship between oral hygiene and periodontitis, whereas, the effect of oral hygiene has been estimated to be stronger than of other established risk factors, such as smoking (16). It may be hypothesized that the socio-behavioral risk factors influence periodontitis occurrence either independently, or through their adverse effect on the oral hygiene status, which in turn raises the probability for the disease establishment.

The present cross-sectional study aimed to examine the periodontal condition of 35-44-year-old Greek adults and assess the relative contribution of socio-demographic characteristics, dental attendance patterns, oral hygiene practices, and smoking habits in the presence of periodontitis, with or without taking into account the oral hygiene level of the adult population.

## Material and Methods

A sample of 1218 35-44-year-old Greek adults was selected, in 2014 individuals being drawn from readily accessible primarily working population groups according to WHO pathfinder survey methodology for the specific age cluster, which ensures the participation of a satisfactory size of people that may present different disease prevalence (17). In order our results to be comparable with previous pathfinder surveys, sample collection has been held in a similar manner and in the same areas as then (18-21). Specifically, the survey covered three urban communities of different socio-economic backgrounds from the two larger metropolitan areas of Greece (Athens and Thessaloniki) and one urban and one rural community from each of five mainland counties, as well as four islands. (in total, 24 sites). Approximately 50 subjects were selected and examined at each site, as this is the standard size for each sampling site recommended as appropriate by the pathfinder sampling methodology established by WHO, in populations with high disease levels (17). In each sampling site, sampling points representing places where 35-44-year-old adults tend to gather were selected randomly (mainly working places in the public and in the private sector, but also places such as churches, culture clubs, public squares, as well as private properties), according to the layout of the region under examination, rural or urban. The average drop-out rate was 20%. It was estimated that the minimum size of the sample to report outcomes of interest, with 95% certainty and an acceptable error of 3%, was 897 subjects. A 30% prevalence of periodontitis was used in this calculation (22), as it referred to the observed in a previous pathfinder survey percentage of 35-44-year-old adults demonstrating at least one tooth site with periodontal pocket depth≥4mm (18).

Calibration of the eight participating examiners took place against a reference examiner that was considered as the ‘gold standard’. Inter-examiner agreement as described by weighted % agreement was estimated to be 85%-96% and 83%-90% for the loss of attachment (LoA) and the Community Periodontal Index (CPI) outcomes, respectively. All examinations were carried out under artificial light using dental mirrors and the WHO CPI periodontal probe. Moisture control was managed by using cotton rolls and sterilized gauze, if needed.

Clinical examination collected data describing gingival bleeding on probing, presence of calculus, periodontal pocketing (pocket depth, PD) and associated loss of periodontal attachment (recorded only if: cemento-enamel junction non-visible and CPI=4, or cemento-enamel junction visible) on teeth 11,31, 16/17, 26/27, 36/37 and 46/47 according to the WHO recommendations (17). Based on the clinical assessment, Community Periodontal Index (CPI) and loss of periodontal attachment (LoA) categorized in multiples of mm (i.e. 0-3mm, 4-5mm, 6-8mm, 9-11mm and 12+mm) were calculated. Furthermore, for the calculation of the simplified oral hygiene index (OHI-S) the proportion of the buccal surfaces of teeth 11, 31, 16 and 26, as well as the lingual surfaces of teeth 36 and 46 covered with soft debris or calculus deposits was recorded. Subsequently, OHI-S was categorized into good (OHI-S= 0.0-1.2), fair (OHI-S=1.3-3.0) or poor (OHI-S=3.1-6.0) (22,23).

Socio-demographic and behavioral data were collected through structured questionnaires. Demographic characteristics (gender, urban/rural location) and socio-economic status parameters (level of education categorized into: up to lower secondary, upper secondary/non-university tertiary, university and income divided into: (≤590€, 591-1200€ and >1200€) were recorded. Periodontal health-related behaviors were described by dental attendance pattern (dental visit when in pain or other emergencies/for treatment/for a check-up and preventive procedures), the time elapsed since last dental visit (up to one year/more than a year), toothbrushing frequency (less than twice per day/twice per day), use of dental floss (no/yes), use of interdental brush (no/yes) and tobacco use (no/1-10 cigarettes per day/>10 cigarettes per day).

Regarding missing data, no correction for non-participation or non-response was applied. Descriptive analysis of CPI, and LoA categories by each socio-demographic/behavioral variable was performed with the use of non-parametric tests (Mann-Whitney and Kruskal-Wallis tests). Univariable and multivariable logistic regression models were used to identify the socio-demographic, behavioral, and clinical indicators for the presence of at least one tooth site with pocket depth≥4mm and with loss of attachment≥4mm. The analysis was performed either by adjusting for the oral hygiene status (OHI-S) of the participants or not, to investigate if the inclusion of a primary clinical indicator attenuates or not the association of the socio-behavioral variables with the periodontal disease outcome. Also, a similar logistic regression analysis was carried out to examine the association between the oral hygiene status of the participants and the socio-behavioral co-variates. In the multivariable analyses, only the univariably significant parameters (*p*≤0.05) were included. Results have been presented with odds ratios (OR) and 95% confidence intervals (CI) with the level of significance set at *p*≤0.05. In all regression analyses the comparisons were estimated against one of the categories that was considered as reference. Data were processed and analyzed with IBM SPSS Statistics (PC version 26.0).

## Results

Descriptive analysis of CPI, and LoA categories is presented in  Table 1, and  Table 2, respectively. The percentage of individuals with healthy periodontal condition was 11.8%, whereas categorization by worst CPI scores revealed 6.4%, 38.6%, 37.4%, and 5.7% of those, presenting with bleeding, calculus, shallow, and deep periodontal pockets, accordingly. Also, the mean number of sextants with a healthy periodontium was 2.0, whereas the corresponding values for shallow and deep periodontal pockets were 1.2 and 0.1, respectively. Furthermore, a mean of 0.1 sextants were excluded from the analysis, as they contained less than two teeth. The analysis of CPI categories by socio-demographic, behavioral and clinical indicators revealed a significantly healthier periodontium among women, urban residents, individuals with higher educational attainment, higher monthly income, frequent dental attenders, non-emergency-oriented dental visitors, dental floss users, better oral hygiene status achievers, non-smokers or non-heavy smokers (≤10 cigarettes per day), and among those who brushed their teeth twice per day.

**Table 1:**
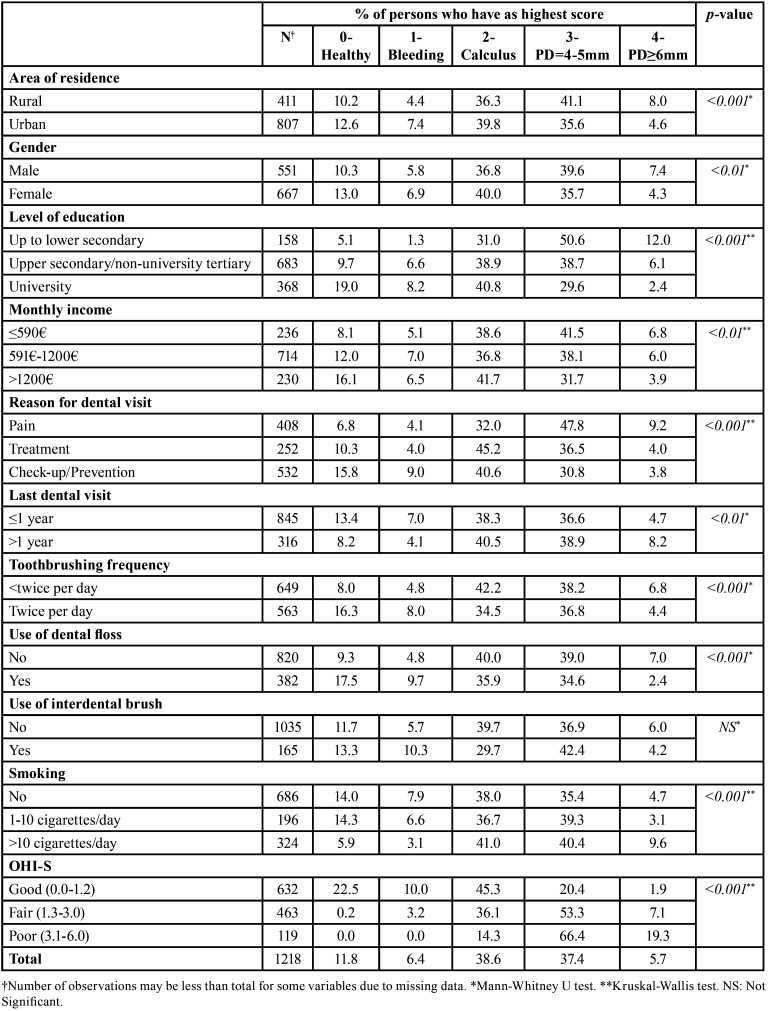
Periodontal conditions of 35-44-year-olds Greek adults measured by CPI, according to socio-demographic, behavioral, and clinical parameters.

**Table 2:**
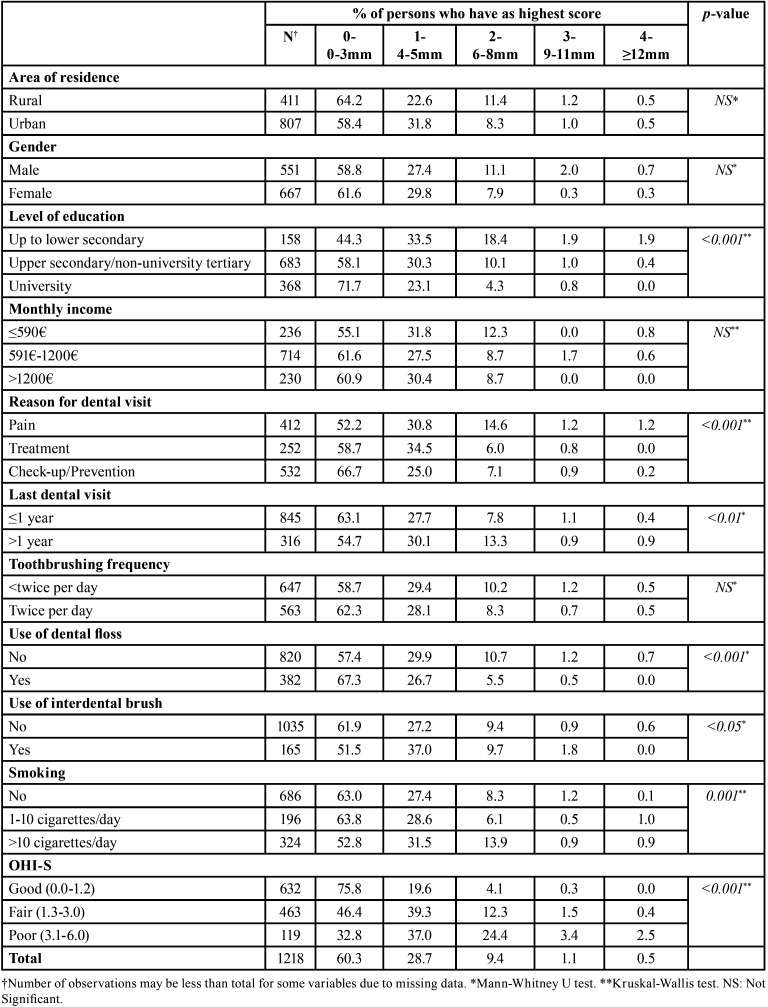
Periodontal destruction of 35-44-year-olds Greek adults measured by Loss of Attachment (LoA), according to socio-demographic, behavioral, and clinical parameters.

The % percentage of subjects with LoA≤3mm was 60.3, whereas respective % percentages for LoA=4-5mm, 6-8mm, 9-11mm and ≥12mm were 28.7, 9.4, 1.1, and 0.5. Also, the majority of sextants presented loss of attachment≤3mm (mean: 4.8), whereas a mean of 0.9 and 0.2 sextants demonstrated loss of attachment 4-5 and ≥6mm, accordingly. The analysis of LoA categories by socio-demographic and behavioral indicators, showed that attachment loss was significantly less common and less severe among higher educated individuals, frequent dental attenders, non-emergency - oriented dental visitors, as well as among participants who used dental floss, did not use an interdental brush, maintained better oral hygiene levels and refrained from tobacco use or smoked up to 10 cigarettes per day.

Univariate analysis of the PD≥4mm distribution (Table 3) showed that all independent variables were significantly related to the outcome, except for the ‘last dental visit’, the ‘toothbrushing frequency’ and the ‘use of interdental brush’ parameters. The unadjusted for the OHI-S multivariable regression model referring to PD≥4mm as the outcome (Table 3) revealed that, a significantly increased probability of presenting at least one tooth with PD≥4mm was linked to lower education attainment and visiting the dentist mainly for emergency treatment. However, in the adjusted for the OHI-S model (Table 3), only emergency-oriented dental visits continued to be significantly independently associated with the presence of PD≥4mm. In the latter model, possessing fair or poor oral hygiene status increased the probability by nearly 4 and 20 times accordingly, of being detected with PD≥4mm.

**Table 3:**
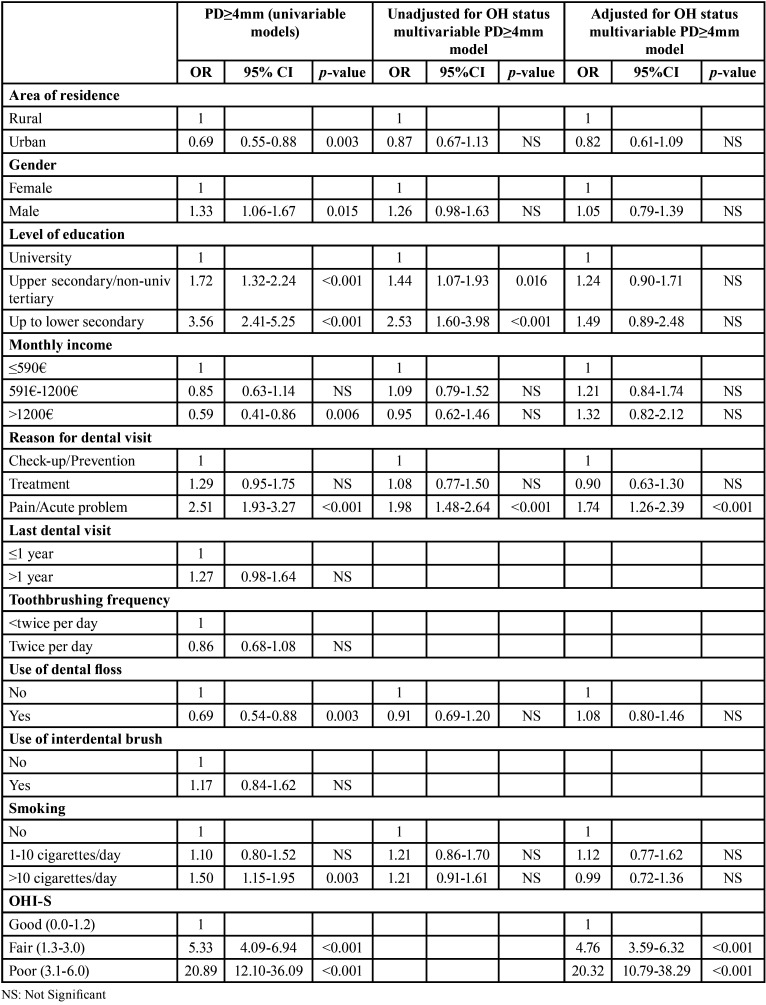
Effect of socio-demographic and behavioral factors on the probability of the presence of at least one tooth with periodontal pocket depth (PD)≥4mm of 35-44-year-old Greek adults a) in univariable logistic regression models and b) in multivariable logistic regression models, either unadjusted (n=1136) or adjusted (n=1132) for the oral hygiene (OH) status of the participants.

In the univariable analysis of the LoA≥4mm distribution (Table 4), only the ‘gender’, the ‘monthly income’ and the ‘toothbrushing frequency’ parameters were not associated with the outcome. The unadjusted for the OHI-S multivariable regression model referring to LoA≥4mm as the outcome (Table 4) revealed that LoA≥4mm probability was significantly independently associated with urban location, lower education attainment, emergency-oriented dental visits, not using dental floss, using an interdental brush and smoking >10 cigarettes per day. In the adjusted for the OHI-S regression analysis (Table 4), only the location, the education level and the use of interdental brush indicators demonstrated a significant independent association. In the latter model, achieving fair or poor oral hygiene status increased the probability by 2.3 and 5.0 times respectively of being detected with LoA≥4mm.

**Table 4:**
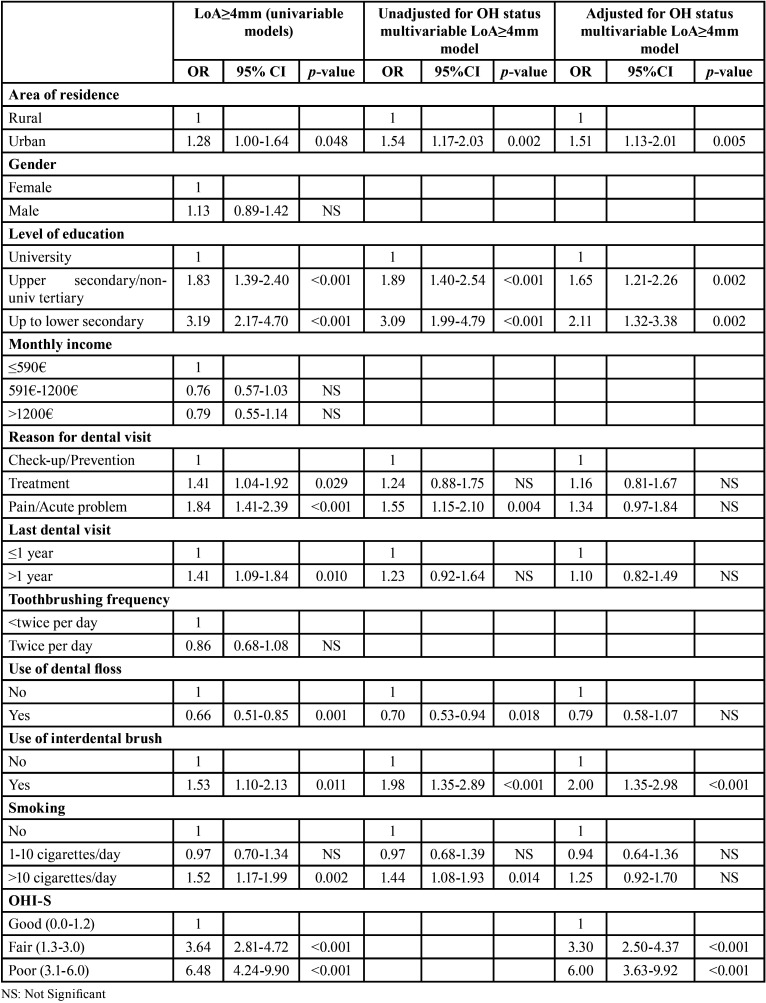
Effect of socio-demographic and behavioral factors on the probability of the presence of at least one tooth with attachment loss (LoA)≥4mm of 35-44-year-old Greek adults a) in univariable logistic regression models and b) in multivariable logistic regression models, either unadjusted (n=1116) or adjusted (n=1113) for the oral hygiene (OH) status of the participants.

Considering the oral hygiene status of the participants, about half of the 35-44-year-olds (52.1%) had good oral hygiene, 38.1% fair, and 9.8% poor. Male gender (*p*=0.001), education up to lower secondary (*p*<0.001) or upper secondary/non university tertiary level (*p*=0.011), monthly income <590 euros (*p*<0.001) or between 590 and 1200 euros (*p*=0.008), infrequent (>1 year) (*p*=0.013) or emergency treatment-oriented (*p*=0.016) dental attendance pattern, toothbrushing <2 times per day (*p*=0.012), not flossing (*p*=0.009) and heavy smoking (>10 cigarettes per day) (*p*=0.001) were significantly independently linked to worse levels of oral hygiene.

## Discussion

The prevalence of periodontal disease observed in Greek 35-44-year-old adults is in line with analogous evidence from other European countries. In Western Europe, among 35-44-year-old adults, the pr evalence of periodontal disease was estimated for PD≥4mm between 12.6% in Sweden (2003) and 73.2% in Germany (2005) whereas for LoA≥4mm between 20.0% in Denmark (2001) and 83.9% in Germany (2005) (8). Regarding the trends in the disease occurrence, a declining pattern could be demonstrated in the UK as the proportion of 35-44-year-old adults who manifested PD≥4mm decreased from 59% to 43% between 1998 and 2009 (8) and in Germany, as the average number of teeth per adult with PD≥4mm diminished from 6.3 to 4.8 between 2005-2014 (4). However, in Greece, the proportion of 35-44-year-old adults presenting shallow or deep pocketing increased from 24.2% to 37.3% and from 3.3% to 5.6%, respectively, between 2005 and 2014 (18).

In the present study, a strong association between oral hygiene levels and periodontitis was observed, as fair and poor oral hygiene increased the probability of PD≥4mm presence by 3.8 and 19.3 times and of LoA≥4mm presence by 2.3 and 5.0 times, respectively, compared with good oral hygiene. These observations accord with recent meta-analysis evidence, where fair and poor oral hygiene were found to significantly increase the risk of having periodontitis by two- and five-fold, accordingly, compared with good oral hygiene (16).

In the adjusted for the OHI-S level of the 35-44-year-old adults, models, different covariates remained independently significant. Specifically, ‘emergency-oriented dental visits’ were associated with PD≥4mm outcome, whereas ‘urban location’, ‘low education level’ and ‘using an interdental brush’ were associated with LoA≥4mm outcome. Seeking dental services primarily when in pain has been associated with worse periodontal health, and it has been suggested that check-up dental visits on a regular basis help to maintain periodontal health and control disease progression (14). Rurality has been reported as a potential risk factor for periodontal disease (8,11). However, as the multilevel nature of the area effect cannot place it as an all-suitable factor when it comes to health outcomes (10), it might be that urban-related environmental stress contributed adversely to periodontal health status. Low education level has been consistently associated with an increased probability for periodontitis (10), and the present study confirmed this observation. Although in prospective studies, interdental brushing was not associated with changes in periodontal pocketing (24), in the present survey LoA≥4mm was significantly higher among participants who used an interdental brush. This can be considered as an elaborate oral health behavior, presumably requiring high oral health literacy; therefore, it appears possible that the minority group (14%) who adopted that habit, mostly included people that were already undergoing periodontally-related treatment, and thereupon were instructed to do so.

Several variables, specifically, ‘lower education level’ for the PD≥4mm model, as well as ‘urgent-oriented dental visits’, ‘not flossing’ and ‘smoking more than 10 cigarettes/day’ for the LoA≥4mm model, were significantly independently associated with periodontitis presence only in the un-adjusted for the OHI-S regression analysis, whereas their association with the respective PD≥4mm or LoA≥4mm outcomes attenuated in the fully adjusted models. As adults with lower education attainment, those who paid dental visits primarily when in pain, did not floss, or smoked heavily demonstrated worse oral hygiene levels, it is reasonable to suggest that oral hygiene status had a mediating effect in the association between these parameters and each respective outcome.

The present study is based on the presentation and analysis of cross-sectional data therefore, causal relationships cannot be drawn from our results. Also, on account of the fact that the simplified sampling methodology developed by WHO has been followed (17), our sample, albeit large, cannot be characterized as random. Nevertheless, it may be regarded as illustrative of the whole population of the 35-44 age cluster, since it ensures the participation of a satisfactory size of people living in representative urban and rural areas of Greece and additionally, it offers the necessary comparability with previous studies conducted likewise. Furthermore, a pragmatic instead of a case definition of periodontitis was utilized, because of the nature of reporting the clinical assessment which was gathered for surveillance purposes.

In conclusion, oral hygiene level appears to play a key role in the periodontal status of 35-44-year-old adults, as fair and poor oral hygiene were the strongest indicators of periodontitis presence. Various socio-behavioural factors may contribute to inadequate oral hygiene maintenance, leading to periodontitis manifestations.
